# Rapid-Acting Treatments for Perinatal Depression: Clinical Landscapes and Future Horizons

**DOI:** 10.3390/ijerph22040546

**Published:** 2025-04-02

**Authors:** Emily M. Beydler, Amanda Koire, Elizabeth Steuber, Joseph J. Taylor, Reid J. Mergler

**Affiliations:** 1 Department of Psychiatry, University of Pennsylvania, Philadelphia, PA 19104, USA; emily.beydler@pennmedicine.upenn.edu; 2 Penn Center for Women’s Behavioral Wellness, University of Pennsylvania, Philadelphia, PA 19104, USA; 3 AMC Department of Psychiatry, Mass General Brigham, Boston, MA 02115, USA; akoire@bwh.harvard.edu (A.K.); jtaylor61@bwh.harvard.edu (J.J.T.); 4 Mary Horrigan Connors Center for Women’s Health and Gender Biology, Brigham and Women’s Hospital, Boston, MA 02115, USA; 5 Harvard Medical School, Boston, MA 02115, USA; elizabeth.steuber@childrens.harvard.edu; 6 Department of Psychiatry, Boston Children’s Hospital, Boston, MA 02115, USA; 7 Center for Brain Circuit Therapeutics, Mass General Brigham, Boston, MA 02115, USA

**Keywords:** perinatal depression, transcranial magnetic stimulation, antidepressants, neurosteroids

## Abstract

Perinatal depression affects approximately 1 in 5 women and is the leading cause of maternal mortality in the United States. In addition to evidence-based treatment with antidepressant medications, there has been a push to identify rapid-acting options for pregnant and postpartum individuals. This paper reviews the evidence behind new pharmacological agents (neurosteroids and ketamine) and non-pharmacological approaches (transcranial magnetic stimulation). The paper also highlights the risks and benefits of electroconvulsive therapy and selective serotonin reuptake inhibitors. Based on recent studies and research, the paper provides considerations when prescribing these modalities including: timing of symptom onset, severity of presentation, breastfeeding priorities, prior treatment response and treatment availability and cost.

## 1. Introduction

Perinatal depression is common, affecting 10–20% of women and contributing to 23% of maternal deaths in the United States [1,2]. Antidepressant medications that have been on the market for decades present evidence-based treatment options for severe perinatal depression and have demonstrated long-term safety data. Yet, not all patients respond to initial antidepressant trials, wish to take long-term medication, or are willing to consider medication at all [3,4,5].

In recent years, there has been a notable push toward identifying novel and rapid-acting treatment options for postpartum depression, culminating in the first Food and Drug Administration (FDA) approval of a medication for postpartum depression [6]. Rapid recovery is especially critical during the perinatal period, given that untreated depression has serious consequences for mothers, their children and families, and society as a whole [1]. During pregnancy, untreated depression is associated with maternal distress and increased risk for suicidality, pre-eclampsia, and preterm delivery [1]; furthermore, if depressive symptoms are left untreated in the postpartum, they are associated with risk of suicide, impaired bonding with the newborn, and difficulty with lactation [1]. With these time-sensitive challenges in mind, this commentary highlights recent advances and future directions for accelerating treatment response in perinatal depression.

## 2. Pharmacologic Approaches

In March 2019, the Food and Drug Administration (FDA) granted breakthrough designation to two new antidepressants: brexanolone and esketamine. Brexanolone was approved specifically for postpartum depression while esketamine was approved for treatment-resistant depression and was not studied in peripartum women with depression. These approvals marked the first time in three decades that the FDA approved antidepressants with novel mechanisms of action and rapid-acting effects.

### 2.1. Neurosteroids

Brexanolone (Zulresso) was developed as the first FDA-approved treatment for postpartum depression. As an allopregnanolone neurosteroid modulator of GABA-A, brexanolone offered a novel mechanism of action unique from other antidepressant treatments and a rapid onset of effect within days (Table 1). However, there were logistical and practical challenges to its use; in addition to requiring Risk Evaluation and Mitigation Strategy (REMS) program requirements for prescribers, treatment with brexanolone required a 60 h inpatient IV infusion and 72 h admission for monitoring. Despite its rapid onset and efficacy, sales of brexanolone remained small at around $1.5 million in the last quarter of its use [7]. Sage Therapeutics recently sunset brexanolone, with the drug no longer being offered as of 1 January 2025 [8]. Brexanolone ultimately served as an encouraging starting point for its successor, zuranolone (Zurzuvae), which is an oral synthetic allopregnanolone analog approved in August 2023. Zuranolone acts similarly to brexanolone as a modulator of GABA-A and is compatible with outpatient treatment since it is prescribed to be taken in oral form nightly for 14 days. When administered postpartum, clinical trials demonstrated that a quarter of patients responded by the third day of treatment, half by the eighth day of treatment, and nearly 60% by the end of the treatment course. At 45-day follow-up, 40% of patients were in remission [9]. While the results are highly encouraging, and insurance companies are improving coverage of zuranolone as a first-line treatment without the need for prior authorization, the eligible patient population is fairly narrow.

To meet treatment criteria for zuranolone, depressive symptoms must have emerged between the third trimester of pregnancy up to 4 weeks postpartum, consistent with DSM-5 criteria for ‘peripartum onset’, and treatment must be administered in the first year postpartum. Practically speaking, this means pregnant women with symptom onset in the third trimester must decide whether to delay treatment until after delivery or pursue a different treatment avenue first. Moreover, given that many women experience depressive symptom onset in the first two trimesters or before pregnancy [10], a large portion of patients with perinatal depression are not eligible. Finally, lactating women were not included in clinical trials for zuranolone, and the formal recommendation is to pause breastfeeding during treatment, despite reassuring unpublished data on transmission in breast milk. In all, it is a valuable treatment option to be aware of perinatal depression, but not right for every patient. Future research will hopefully clarify whether zuranolone’s effects can be generalized to symptom onset at other times in the perinatal period and provide more safety data for lactation. To expand access to eligible patients, efforts should focus on removing insurance-specific restrictions; currently, for some insurance plans, prescriber access is limited to psychiatry and there is a requirement to trial other antidepressants first.

It is encouraging that brexanolone and zuranolone’s approval heralded the development of several novel neurosteroids in the clinical trial pipeline for perinatal depression. On the frontier, a third agent, ganaxolone, is currently undergoing phase II clinical trials for postpartum depression (PPD) after it was FDA-approved for seizures associated with cyclin-dependent kinase-like 5 deficiency disorder [11,12]. Ganaxolone is available in IV and oral formulations, and as an allopregnanolone analog, it differs from brexanolone and zuranolone in that its lower affinity for the progesterone receptor allows longer-term use [13]. A fourth drug, NORA-520, is an allopregnanolone pro-drug undergoing phase II clinical trials in the United States (U.S.) [14]. Finally, BRII-296 is another allopregnanolone pro-drug currently in phase II clinical trials in an intramuscular, long-acting injectable formulation [15]. BRII-296 showed sustained plasma levels for 3–4 weeks, making it a potentially accessible and convenient option for patients struggling with daily medication adherence [16].

### 2.2. Ketamine/Esketamine

Ketamine has gained attention for treating treatment-resistant depression and suicidal ideation, but current guidelines do not support its use during pregnancy, mainly due to rodent studies showing negative effects like depression, anxiety, and ADHD-like symptoms in offspring [17]. Despite the lack of safety data, ketamine-exposed pregnancies are likely to increase, as most ketamine clinics in the United States do not discuss pregnancy risks during informed consent. Around 80% do not test for pregnancy before treatment, and 86% do not require contraception [18]. Regarding safety while breastfeeding, a 2023 case series showed that the relative infant dose of ketamine was less than 1%, well below the relative safety threshold for exposure of 10%, though overall data remains limited [19,20].

To date, no clinical trials of perinatal esketamine have been conducted in the United States. Several studies have been conducted in China since 2021 [21], exploring perioperative epidural ketamine use as part of a national effort to improve maternal outcomes and reduce Cesarean section rates through enhanced epidural analgesia [22]. Notably, ketamine is not widely used in epidural analgesia in the United States. Additionally, many of these studies do not mirror how patients are evaluated or diagnosed clinically for postpartum depression in psychiatry. A recent trial studied women with low or no depressive symptoms before delivery yet used day 7 postpartum as the primary endpoint for making a diagnosis of depression using a self-report depression screening tool [23]. However, a two-week duration of symptoms is a basic diagnostic criterion for a diagnosis of depression, and there were no significant differences in depressive symptoms between esketamine-infused epidurals and placebo at two weeks and beyond. Though the trial claimed to prevent postpartum depression, the data more likely points to a reduction in postpartum blues, which lacks clear clinical significance as postpartum blues is a widespread and self-limited phenomenon. However, a recent multicenter randomized clinical trial from Peking University First Hospital in China showed more promising results [24]. The study involved over 300 patients with mild antenatal depression diagnosed by clinical interview who received a single low dose of esketamine epidural analgesia or a placebo shortly after delivery. At 42 days postpartum, major depressive episodes were reduced by 75% in the esketamine versus placebo group. In terms of side effects, those receiving ketamine had mild, transient neuropsychiatric side effects, which did not correlate with depression prevention. The trial excluded participants with pre-pregnancy mood disorders and chose patients with mild antenatal depressive symptoms, limiting its generalizability to patients with more severe mental health conditions.

In short, while this is an interesting area for future research, the administration of esketamine in epidural analgesia in women without depressive symptoms to prevent ‘baby blues’ and potentially PPD is not recommended and does not reflect current epidural analgesia practices in the United States. More research is needed to clarify any potential role in preventing and treating postpartum depression, particularly in those with severe symptoms. The safety of breastfeeding while using esketamine also requires further study.

## 3. Non-Pharmacologic Approaches

Concerns about the transmission of medications in breast milk or across the placenta deter some women from utilizing pharmacotherapy [25]. For those who inquire about rapid-acting nonpharmacologic treatments for perinatal depression, neuromodulation leads the way.

### Transcranial Magnetic Stimulation

Repetitive transcranial magnetic stimulation (TMS) is a notable option for the peripartum period, as there is neither pharmacologic exposure to the fetus in pregnancy nor effect on lactation in the postpartum. TMS involves targeted magnetic pulses repeatedly applied to a specific brain region on the cortical surface. In the case of major depressive disorder, the predominant region targeted is the left dorsolateral prefrontal cortex (lDLPFC). Functional neuroimaging studies have demonstrated anti-correlation between the lDLPFC and subgenual cingulate that decreases after high-frequency TMS treatment and appears to correlate with depressive symptom improvement [26,27].

Though its literature is in its infancy, TMS has shown promise as a safe and acceptable treatment during pregnancy. There are reports of TMS use during all three trimesters with minor risks, including transient discomfort, headache, and supine hypotension [28]. Moreover, the offspring of women treated with TMS while pregnant demonstrated no negative developmental outcomes compared to untreated women with depression when followed up to 62 months [29].

While early, the efficacy of TMS treatment during the peripartum period is promising. The majority of the literature surrounding TMS use in pregnancy is based on case reports, case series, and single-arm trials, which indicate an overall treatment benefit [30]. Only one small randomized controlled trial (n = 11) has examined TMS use during pregnancy [31]. Results demonstrated significant reductions in depression severity ratings with active treatment compared to sham over the one-month treatment time, with reductions in symptoms by approximately 50% across depression measures [30,31]. No statistically significant differences in remission and response rates emerged, which may be attributable to the small trial size. Postpartum data are also sparse, but promising [30,31]. Case studies (n = 2) and single-arm trials (n = 3 trials, 34 total patients) overall show treatment benefits [32,33,34,35,36]. Most of the clinical trial data during the postpartum period is not available in English, limiting critical appraisal. Similarly to the trial during pregnancy, the one trial available in English reported that average Hamilton Depression Rating Scale scores decreased from 29.1 before treatment to 18.4 post-treatment in the active treatment group, corresponding to a decrease from very severe to moderate depression symptoms [37]. However, small sample sizes (n = 8 treatment group, n = 6 sham group) again limited statistical power. Larger studies with robust sham-controlled treatment groups are needed to assess efficacy. However, the safety and tolerability profile appear positive for TMS, and efficacy data warrants cautious optimism.

Notably, the time course for a standard TMS protocol is lengthy, requiring six to eight weeks of daily treatment for at least 30 min for treatment benefit. Fortunately, a shortened protocol, intermittent theta burst stimulation (iTBS), can be completed in approximately three minutes rather than 30–40 min with similar efficacy and safety profiles [38]. To date, there is one existing report of a pregnant patient plateauing during TMS treatment and then further improving with iTBS treatment [39]. Furthermore, there is growing enthusiasm for accelerated iTBS protocols, which have led to robust and rapid decrements in depression symptoms in clinical trials without an increased risk profile in individuals with major depressive disorder. Stanford Accelerated Intelligent Neuromodulation Therapy (SAINT) is perhaps the most well-known of these protocols, administering 50 image-guided treatments over five days. The initial results demonstrated a 90.5% remission rate of patients who completed treatment over five days and a 60% remission rate at one-month follow-up [40]. The only double-blind, randomized controlled trial of SAINT was discontinued after an interim analysis (n = 29) revealed a large effect size (Cohen’s d > 0.8) for active vs. sham (52.5% vs. 11.1% MADRS reduction, respectively). SAINT is FDA-cleared but not widely available, and questions remain about the necessity of imaging guidance.

Accelerated protocols deploy a higher “dose” of treatment in a shorter amount of time, which may contribute to the treatment benefit. Given its efficacy in treatment-resistant depression, accelerated protocols may hold promise for the treatment of perinatal depression. One case report presents data on its use during peripartum [33]. The individual received five daily treatments of iTBS for five days, followed by weekly sessions for five weeks. Montgomery Asberg depression ratings decreased from 21 to 14 in two weeks, corresponding to a decrease from moderate to mild depressive symptoms. Symptoms further decreased by another 50% between the conclusion of treatment and five weeks post-treatment. To date, one additional study has deployed accelerated iTBS postpartum. In a study of 32 women with postpartum depression, depressive symptom severity significantly improved over the course of treatment, and changes in functional connectivity in circuits involving the salience network and default mode network were observed after treatment [41]. Accelerated iTBS is a particularly exciting new avenue of clinical intervention, given the significant efficacy seen in treatment-resistant depression and the rapidity of improvement [42].

## 4. Revisiting Old Strategies

Although the new treatments hold promise for rapid-acting and effective ways to help our patients with depression in the peripartum, it is critical to highlight when the use of more established agents is appropriate.

### 4.1. Electroconvulsive Therapy (ECT)

For severe depressive symptoms in the peripartum period, both the American Psychiatric Association (APA) and the American College of Obstetricians and Gynecologists endorse the use of ECT, and note its safety and efficacy during all trimesters of pregnancy [43]. According to a recent systematic review of case reports of 130 women treated with ECT during all trimesters of pregnancy, ECT is safe and effective throughout pregnancy in the majority of cases, with most adverse events being low- and moderate-grade. The most common complication, reported in 10 cases, was fetal arrhythmias, which resolved spontaneously without medical intervention [44]. Additionally, ECT techniques have evolved over the years, and this has further increased its degree of safety [44]. The rates of adverse outcomes, including preterm delivery and miscarriage, have not differed from those in the general public, and there do not appear to be congenital defects or neuropsychological sequelae for the offspring of mothers treated with ECT [45].

For pregnant patients, symptoms of transient uterine contractions occur in about 27% of cases, while premature labor is reported in 4–11% of cases [46]. Fetal side effects are typically limited to transient bradycardia, with no long-term impact on growth. Additionally, the time course for a full trial is lengthy, lasting around 4 weeks for 12 sessions two to three times weekly in the initial index series, with the minimum effective number of sessions in past studies found to be between 6 and 12 for patients [47,48]. Despite these drawbacks, ECT appears to be a viable treatment option in severe, life-threatening, or treatment-refractory cases of perinatal depression.

### 4.2. Selective Serotonin Reuptake Inhibitors (SSRIs)

One impetus for fast-acting treatments for perinatal depression is the perception that more conventionally prescribed antidepressants take too long to work and are not always effective. While SSRIs typically require six to eight weeks [49] to be considered a full trial, data shows responders often experience noticeable symptom improvement within two to four weeks [50,51,52,53,54]. Caution about fetal medication exposure may lead to prolonged uptitrations of medications even when the patient has not been responding to the medication in the early months of treatment. Given reassuring safety data for SSRIs, these findings encourage less hesitation around switching when the goal is to prioritize maternal stability. Based on several studies, SSRIs are not associated with an increased risk of major or minor congenital malformations, and thus, they are not considered teratogenic [4]. It is critical that psychiatrists have discussions with pregnant individuals about the risks of starting a medication versus the risks of stopping and recurrence of anxiety or depression during pregnancy. In the postpartum, lactation is also a consideration, as psychotropic medications can be transmitted, through breastmilk in varying degrees though use is generally considered compatible with lactation.

While for-profit companies offer pharmacogenomic testing that claims to guide medication choices towards those more likely to be effective, which would theoretically speed recovery and reduce fetal exposures, the evidence does not support clinical benefit, and the American Psychiatric Association (APA) does not recommend its use for treating depression [55]. The International Society of Psychiatric Genetics has summarized pharmacogenomic evidence and guidelines for antidepressants and does not recommend it as an approach to treatment selection, though it does note potential utility in clarifying dosing needs if patients are not responding to typical dose ranges and are found to be slow or rapid metabolizers of medications. It is important to note that the testing is not regulated, and many of the available tests include genes that have little to no support for clinical implementation. Moreover, the United States Food and Drug Administration cautions against the use of pharmacogenomic testing to guide antidepressant prescribing [56].

## 5. Considerations for Treatment Selection

The emergence of neurosteroid treatments represents a significant advance, particularly in offering rapid relief for postpartum depression. However, SSRIs remain appropriate first-line treatments for many patients, particularly those with symptoms emerging before the third trimester or more than 4 weeks postpartum, who would not qualify for zuranolone therapy (Table 2).

The treatment landscape for perinatal depression has evolved significantly with these newer options. When considering which treatment to recommend, the following applies:

1. Timing of symptom onset: For symptoms beginning in the third trimester or within 4 weeks postpartum, zuranolone presents a rapid-acting option with good efficacy.
2. Severity of presentation: For severe, life-threatening depression, ECT remains the gold standard despite its stigma. Accelerated TMS protocols show promise as an alternative rapid-acting option without medication exposure.
3. Breastfeeding priorities: SSRIs generally offer the best-established safety profile for lactating women. Neurosteroids currently require temporary cessation of breastfeeding, though this guidance may evolve as more data becomes available.
4. Prior treatment response: For patients with histories of SSRI non-response, newer options like neurosteroids or neuromodulation may be particularly valuable.
5. Treatment availability and cost: Many of the rapid-acting treatments (particularly brexanolone and accelerated TMS protocols) have limited availability and/or high costs, which must be factored into treatment planning.

## 6. Conclusions

The field of reproductive psychiatry is undergoing a transformative era, with novel treatments like neurosteroids, ketamine, and neuromodulation offering rapid-acting solutions to peripartum depression. These advancements provide much-needed options to address the urgent risks associated with untreated maternal mental health conditions. While SSRIs remain effective for many patients, and ECT remains the gold standard for severe cases, these innovations significantly expand the therapeutic toolkit.

However, the accessibility of these treatments poses a significant challenge. Delays in obtaining care due to systemic barriers, such as long wait times and community obstetrician and psychiatrist shortages, can negate the benefits of rapid-acting treatments. Given that it is not mandatory to teach reproductive psychiatry to psychiatry residents in the United States, this can contribute to future ongoing challenges with access to psychiatrists skilled in treating pregnant and postpartum individuals [57]. Each month of delayed treatment increases the risk of symptom worsening, suicidal ideation, and maternal distress [10]. Bridging this gap requires expanding access to qualified reproductive psychiatric care in both academic and community settings.

As the field continues to innovate, addressing these systemic barriers will be essential to translating clinical advancements into meaningful improvements in population health. By combining evidence-driven treatment approaches with a commitment to equitable access, we can ensure that every patient has the chance to benefit from this exciting new era in perinatal psychiatry.

## Figures and Tables

**Table 1:** Pharmacokinetic properties, clinical efficacy, treatment duration, and safety profiles of treatments for perinatal depression.

Treatment	Mechanism of Action	Pharmacology	Onset of Action	Treatment Duration	Response Rate	Remission Rate	Pregnancy Safety	Lactation Compatibility	Special Considerations
Zuranolone	Allopregnanolone analog; GABA-A positive allosteric modulator	Oral; 14-day course	Within days	14 days	25% response by day 3; 50% by day 8, ~60% by end of treatment	~40% at 45-day follow-up	Limited data;	Recommended to pause breastfeeding; limited data	Only for symptoms emerging in 3rd trimester—within 4 weeks postpartum; must be used within 1 year postpartum
Brexanolone	Allopregnanolone; GABA-A positive allosteric modulator	IV infusion over 60 h	Within days	60 h infusion; 72 h monitoring	Superior to placebo in clinical trials	Superior to placebo in clinical trials	Limited data	Limited data	Discontinued as of Jan 2025; required inpatient administration
SSRIs	Serotonin reuptake inhibition	Oral; daily dosing	Response at 2–4 weeks, full trial of 6–8 weeks	Months to years	Variable	30–50% typically	Generally safe; not associated with increased risk of major congenital malformations	Variable transmission in breast milk; generally safe	Extensive long-term safety data available
ECT	Induces generalized seizure; affects multiple neurotransmitter systems	Procedure performed 2–3 times weekly	6–12 sessions (2–4 weeks)	4+ weeks	~70% for severe MDD	~50–60% for severe MDD	Safe in all trimesters	Safe	Temporary memory impairment
Repetitive TMS & iTBS	Modulates neural activity via magnetic pulses	3–40 min sessions, 5 days/week	Weeks	6–8 weeks	Limited data; promising in small studies	Limited peripartum data	Likely safe; minor risks (e.g., headache, low seizure risk)	Likely safe	No medication exposure; requires significant time commitment
Accelerated iTBS	Modulates neural activity via magnetic pulses	3–10 min sessions, multiple times daily	Days to weeks	5 days (intensive)	Limited data; promising in small studies	Limited peripartum data	Likely safe based on standard TMS data	Likely safe	Limited availability; emerging but promising option
Ketamine/Esketamine	NMDA receptor antagonist	IV or intranasal administration	Hours to days	Single dose to repeated administration	Limited data; 75% reduction in MDD episodes	Limited peripartum data	Not recommended on animal studies	<1% relative infant dose in breast milk (limited data)	Concerns about offspring neurodevelopment; data emerging
Ganaxolone (investigational)	Allopregnanolone analog; GABA-A modulator	Oral or IV administration; longer half-life	Unknown	Unknown	Phase II trials underway	Phase II trials underway	Unknown	Unknown	Lower progesterone receptor affinity; may allow longer-term use
NORA-520 (investigational)	Allopregnanolone prodrug	Oral administration	Unknown	Unknown	Phase II trials underway	Phase II trials underway	Unknown	Unknown	Limited data available
BRII-296 (investigational)	Allopregnanolone prodrug	Long-acting injectable; sustained levels for 3–4 weeks	Unknown	Single injection lasts 3–4 weeks	Phase II trials underway	Phase II trials underway	Unknown	Unknown	May improve medication adherence due to injectable formulation

**Table 2:** Comparative advantages and disadvantages of treatments for perinatal depression.

Treatment	Key Advantages	Key Disadvantages	Best Suited For
Zuranolone	Rapid onset; oral administration; outpatient treatment	Narrow eligibility window; recommendation to pause breastfeeding; high cost	Patients with symptom onset in 3rd trimester or within 4 weeks postpartum requiring rapid response
SSRIs	Extensive safety data; compatible with breastfeeding; affordable	Slower onset of action; daily administration required	First-line for mild to moderate perinatal depression when rapid response not critical
ECT	High efficacy; safe throughout pregnancy; rapid for severe cases	Requires medical facility; stigma; temporary cognitive effects	Severe, life-threatening, or treatment-resistant depression
TMS (Standard)	No medication exposure; safe in pregnancy and lactation	Time-intensive; limited availability; slower response	Patients unwilling/unable to take medication with moderate symptoms
iTBS (Accelerated)	Rapid response; no medication exposure; brief sessions	Very limited availability; emerging evidence	Moderate to severe symptoms requiring rapid response without medication
Ketamine/Esketamine	Potentially rapid response	Safety concerns during pregnancy; limited peripartum data	Not currently recommended for routine use in perinatal depression
